# Healthcare professionals’ attitudes to mandatory COVID-19 vaccination: Cross-sectional survey data from four European countries

**DOI:** 10.1080/21645515.2023.2256442

**Published:** 2023-09-19

**Authors:** Linda C. Karlsson, Amanda Garrison, Dawn Holford, Angelo Fasce, Stephan Lewandowsky, Frederike Taubert, Philipp Schmid, Cornelia Betsch, Fernanda Rodrigues, Lisa Fressard, Pierre Verger, Anna Soveri

**Affiliations:** a Department of Clinical Medicine, University of Turku, Turku, Finland; b Faculté des Sciences Médicales et Paramédicales, Southeastern Health Regional Observatory (Observatoire Régional de la Santé, ORS-PACA), Marseille, France; c School of Psychological Science, University of Bristol, Bristol, UK; d Faculty of Medicine, University of Coimbra, Coimbra, Portugal; e Department of Psychology, University of Potsdam, Potsdam, Germany; f School of Psychological Science, University of Western Australia, Perth, Australia; g Institute for Planetary Health Behavior, Health Communication, University of Erfurt, Erfurt, Germany; h Health Communication Working Group, Implementation Research, Bernhard Nocht Institute for Tropical Medicine, University of Hamburg, Hamburg, Germany; i Centre for Language Studies, Radboud University Nijmegen, Nijmegen, The Netherlands

**Keywords:** Vaccines, mandates, attitudes, COVID-19, healthcare professionals, HCPs, Europe, compulsory vaccination, reactance

## Abstract

Mandatory vaccinations are widely debated since they restrict individuals’ autonomy in their health decisions. As healthcare professionals (HCPs) are a common target group of vaccine mandates, and also form a link between vaccination policies and the public, understanding their attitudes toward vaccine mandates is important. The present study investigated physicians’ attitudes to COVID-19 vaccine mandates in four European countries: Finland, France, Germany, and Portugal. An electronic survey assessing attitudes to COVID-19 vaccine mandates and general vaccination attitudes (e.g. perceived vaccine safety, trust in health authorities, and openness to patients) was sent to physicians in the spring of 2022. A total of 2796 physicians responded. Across all countries, 78% of the physicians were in favor of COVID-19 vaccine mandates for HCPs, 49% favored COVID-19 vaccine mandates for the public, and 67% endorsed COVID-19 health passes. Notable differences were observed between countries, with attitudes to mandates found to be more positive in countries where the mandate, or similar mandates, were in effect. The associations between attitudes to mandates and general vaccination attitudes were mostly small to neglectable and differed between countries. Nevertheless, physicians with more positive mandate attitudes perceived vaccines as more beneficial (in Finland and France) and had greater trust in medical authorities (in France and Germany). The present study contributes to the body of research within social and behavioral sciences that support evidence-based vaccination policymaking.

## Introduction

Vaccine-preventable diseases continue to contribute to increased morbidity and mortality, even in high-income countries where vaccines are easily accessible. A reason for this is suboptimal uptake of many vaccines,^1^ which limits the protective benefits of those vaccines because an insufficiently high proportion of the population is vaccinated.^2,3^ To increase vaccine uptake and halt disease transmission among the public and at-risk groups, several national authorities have implemented mandatory vaccination policies.^4–6^ Healthcare professionals (HCPs) are frequently the target of vaccine mandates because they have an increased risk of exposure to infectious diseases, are in close contact with vulnerable individuals, and play a critical role in maintaining the delivery of healthcare.^7–10^ In 2018, 13 of 36 European countries (36%) implemented mandatory vaccination policies for HCPs.^11^ In some countries, vaccination is a requirement for HCPs’ employment, whereas, in others, unvaccinated HCPs might receive a fine, be moved to low-risk tasks, or have their employment terminated.^11^

Vaccine mandates are not universally accepted because they restrict individuals’ freedom to make their own health decisions. Debates on mandates often revolve around the right to personal autonomy, individual or professional obligations, the effectiveness of mandates, and the social and psychological consequences that mandates may have.^9,10,12^ Due to the complexity of the issue, decisions around the appropriateness, necessity, feasibility, and successful implementation of mandates, require careful ethical and practical evaluation.^13,14^ This includes assessing the targeted population’s receptivity and attitudes to mandates. As mandatory vaccination policies for HCPs are becoming more common,^11^ it is increasingly important to understand HCPs’ perceptions about these mandates. In addition, HCPs form a crucial link between vaccination policies and the public and are considered the most trustworthy source of vaccine-related information.^15,16^ Because HCPs often need to communicate the importance of vaccination to their patients, it is relevant to understand their opinions on vaccine mandates for the public. Furthermore, it is essential to ensure that HCPs remain evidence-based and to prevent ill-designed policies from causing HCPs to feel anger or reactance that could override their existing vaccination attitudes. In the present study, we aim to support decision-making related to potential mandate implementation by investigating attitudes to vaccine mandates among physicians in four European countries with different vaccine mandate traditions; Finland, France, Germany, and Portugal.

During the pandemic, several European countries mandated COVID-19 vaccination in different ways and for different target groups.^7,8^ Some countries required HCPs to get vaccinated against COVID-19 (e.g., Italy, France, Germany, and Greece) and some introduced a “health pass” which made access to certain public spaces available only to members of the public with proof of vaccination, a negative COVID-19 test result, or recent recovery from COVID-19 infection (e.g., Italy, France, Germany, and Portugal). Mandatory COVID-19 vaccines for members of the public were rare, although the general population in Austria, and individuals belonging to risk groups in Greece, were required to get vaccinated for COVID-19.^7,8^

The attitudes of HCPs toward mandatory COVID-19 vaccination have not been widely documented, and the existing literature has found varying acceptance rates for the mandates. In 2021, the proportion of HCPs who reported being in favor of mandatory COVID-19 vaccination policies (without a specific target group for the mandate) was 34% in Cyprus and 38% in the US.^17,18^ When it comes to mandates specifically for HCPs, HCPs report higher support. In a study of US medical students, 58% agreed that COVID-19 vaccines should be mandatory for HCPs, whereas only 16% supported mandates for the public.^19^ In a study conducted in India of medical students at the beginning of 2021, 75% agreed that COVID-19 vaccines should be mandatory for HCPs, whereas 66% thought that the vaccines should be mandatory for people traveling within India.^20^

Research conducted prior to the pandemic on HCPs’ attitudes to vaccine mandates also shows substantial variation across studies. Part of this variation might be due to social, political, and cultural differences between the countries in which the studies were conducted, as well as differences in vaccine access. A meta-analysis of 40 studies conducted among HCPs in Europe, Asia, America, and Oceania found that the rates of HCPs agreeing with mandatory influenza vaccination policies for HCPs varied between 15% and 93% across studies.^21^ Previous research has also documented individual-level factors, such as vaccine attitudes, that are associated with HCPs’ attitudes to vaccine mandates. In these studies, HCPs with higher confidence in vaccines, greater trust in official sources of vaccine information, larger perceived collective responsibility, and lower complacency report higher acceptance of mandate policies.^22,23^ When it comes to COVID-19, HCPs who support COVID-19 vaccine mandates have been found to report greater trust in health authorities, the healthcare system, and the effectiveness of the COVID-19 vaccine, as well as greater general vaccination knowledge.^17^ The present study contributes to this body of literature by assessing the relationship between vaccination attitudes and attitudes toward vaccine mandates among HCPs in different countries. To the best of our knowledge, the present study is the first to investigate this relationship in several countries simultaneously, using a measure that covers a broad range of vaccination attitudes and that has been validated in each country. This enables a reliable cross-country evaluation of the role of vaccination attitudes in HCPs’ attitudes to mandates.

### Objectives of the current study

In the present study, we investigated physicians’ attitudes to mandate policies for COVID-19 vaccines in four European countries: Finland, France, Germany, and Portugal. The objectives were to investigate 1) the extent to which physicians are favorable to vaccine mandates, 2) whether physicians’ attitudes to vaccine mandates vary between countries, and 3) whether physicians’ attitudes to vaccine mandates are associated with their vaccination attitudes and vaccine recommendation behaviors. We examined attitudes to COVID-19 vaccine mandates for HCPs, attitudes to COVID-19 vaccine mandates for the public, and attitudes to COVID-19 health pass policies. For vaccination attitudes, we assessed a range of attitudes concerning vaccines and vaccine-related work. This encompassed perceived vaccine risks, perceived benefit-risk balance of vaccines, perceived collective responsibility, trust in authorities, commitment to vaccination, self-efficacy, openness to patients, perceived constraints, and whether the HCP trusts the vaccination system and recommends vaccines despite potential concerns.

The investigated countries shared some similarities in implementing COVID-19 vaccine mandates. None of the countries applied mandatory COVID-19 vaccination for the public during the pandemic, but all implemented health pass policies. COVID-19 vaccines were mandated for HCPs in Finland, France, and Germany, but not in Portugal, and these mandates were in effect during the time of data collection. There were more differences between the four countries regarding mandates for vaccines other than COVID-19. In Finland, no vaccines are mandated for the general public, but HCPs working with risk groups are required by law to be immunized against measles, pertussis, varicella, and influenza, otherwise they can be moved to other tasks.^24^ In France, 11 vaccines are mandated to the public, of which eight became mandatory as recently as 2018 in an effort to address suboptimal vaccine uptake.^25^ In addition, French HCPs are required to get vaccinated against hepatitis B. Germany has also recently introduced mandatory vaccination policies for the public: as of 2020, these policies require children to be immunized against measles to attend daycare and school, and adults working in medical facilities, daycare, schools, and refugee accommodations are also required to be immunized.^26^ In Portugal, there are no mandatory vaccinations for HCPs or the public. An overview of mandates in effect at the time of data collection in each country is presented in Table 1.

**Table 1.** Mandates in effect at the time of data collection by country.

	COVID-19 mandates			Other mandates		
Country	Public	HCPs	Health pass	Public	HCPs	Health pass
Finland	No	Yes	Yes	No	Yes	No
France	No	Yes	Yes	Yes	Yes	No
Portugal	No	No	Yes	No	No	No
Germany	No	Yes	Yes	Yes	Yes	No

Public = Vaccines mandated for members of the general public. HCPs = Vaccines mandated for (groups of) healthcare professionals. Health pass = Proof of vaccination, negative test result, or recovery required to access certain public spaces. Yes = Mandate in effect. No = Mandate not in effect.

## Materials and methods

### Study population and procedure

The data were collected using a cross-sectional survey and individuals were recruited through a self-selection sampling approach. Physicians in Finland, France, Germany, and Portugal were invited to fill out an electronic, self-administered questionnaire. Recruitment channels (e-mail list or panel provider) and types of physicians targeted differed between the countries, as different types of physicians are responsible for or actively involved in vaccinations in each country. General practitioners (GPs) were targeted in France, whereas Finland and Portugal targeted GPs and pediatricians. In Germany, GPs, pediatricians, and gynecologists were targeted. Information about recruitment in each country is presented in Table S1. Data collections took place between March and May 2022. The study was approved by the Ethics Committee for Human Sciences at the University of Turku in Finland (reference: 1/2022), the Ethics Committee of Aix-Marseille University in France (reference: 2021-12-16-01), the Ethics Committee of the University of Erfurt in Germany (reference: 20210713), and the Ethics Committee of the Faculty of Medicine at the University of Coimbra in Portugal (reference: 093-CE-2021). All respondents gave their informed consent to participate in the study. Consent was provided electronically at the start of the survey. The data collection methods, including target populations, expected sample size, and inclusion criteria, were preregistered in Finland, Germany, and Portugal before collecting the data (Table S1).

We determined sample size targets based on highly conservative assumptions (simple random sampling, estimating a percentage around 50% for binary outcome variables, confidence interval of ±5%). Power analysis indicated that a sample of *N* = 400 would be sufficient for a 95% confidence interval of [45%–55%].

### Measures

The current study was part of a larger survey. In addition to the measures included here, the survey contained questions on other topics, such as attitudes to complementary and alternative medicine as well as perceived difficulty of rebutting different anti-vaccination arguments. Analyses related to these measures are described in other publications.^27,28^ Only the measures analyzed for the present study are described below. The exact wording of the measures can be found in Table S2. All questions were translated from English to the target language by two professional translators, then a consensus version of the questionnaire was back-translated to English by two additional professional translators. To examine the meaningfulness and appropriateness of the survey questions within each cultural context and to ensure the items were interpreted in the way intended, cognitive interviews were carried out for each language version following the guidelines by Peterson et al.^29^ In each country, between five and seven GPs or pediatricians were interviewed. The survey was also pilot tested in each country (total *N* = 207). For a detailed description of the survey development process, please see Garrison et al.^30^ The median time to complete the whole survey was 14 minutes.

#### Attitudes to COVID-19 mandates

The questionnaire included three items measuring attitudes to COVID-19 mandates. The items consisted of statements about whether respondents thought COVID-19 vaccines should be mandatory for HCPs in their country, whether they thought the vaccines should be mandatory for the general public in their country, and whether they agreed with the use of a COVID-19 health pass, respectively. Physicians responded on a scale from 1 (*strongly disagree*) to 5 (*strongly agree*) with the middle response alternative labeled *undecided*. The health pass item was not administered in Germany because several types of health passes were applied in the country at the time of data collection. Administering several questions related to different health passes was not considered optimal for the length of the questionnaire and comparability between countries.

#### Attitudes to vaccinations

Vaccination attitudes were assessed using the I-Pro-VC-Be questionnaire.^30,31^ The tool consists of 33 items that measure 10 different constructs that relate to HCPs’ attitudes to vaccination: perceived vaccine risks (i.e., how safe HCPs perceive certain vaccines to be), complacency (i.e., how useful HCPs perceive vaccines to be), perceived benefit-risk balance of vaccines (i.e., the degree to which HCPs perceive that the benefits of vaccines outweigh the risks), perceived collective responsibility (i.e., the extent to which HCPs recommend vaccines to contribute to community immunity), trust in authorities (i.e., trust in relevant institutions to provide reliable vaccine information and to define the vaccination strategy), commitment to vaccination (i.e., the extent to which HCPs are proactive in motivating their patients to accept vaccinations), self-efficacy (i.e., how prepared HCPs feel in terms of knowledge and skills to address vaccination with patients), openness to patients (i.e., attitudes toward [hesitant] patients), perceived constraints (i.e., perceived practical constraints to vaccination), and reluctant trust (i.e., the extent to which HCPs trust the vaccination system and recommend vaccines despite potential concerns). The I-Pro-VC-Be is an international adaptation of the Pro-VC-Be tool originally developed for French-speaking contexts.^31,32^ The Pro-VC-Be is based on three theoretical frameworks, including the Theoretical Domain Framework^33,34^ (a consensus approach based on a combination of theories of behavior and behavioral change providing relevant constructs for the development of evidence-based practices for HCPs), the Health Belief Model^35,36^ (which describes predictors of health-related behaviors), and the 5C model^37^ (which describes psychological antecedents of vaccination behaviors in the general public). A previous study has validated the tool in the sample included in the present study and established measurement invariance, meaning that the physicians’ responses can be reliably compared across countries.^30^ In addition to the vaccination-attitude constructs in the I-Pro-VC-Be, the questionnaire included two items measuring the degree to which the physicians perceive the professional norms among their colleagues in their country to be in favor of vaccination. The response scale ranged from 1 (*strongly disagree*) to 5 (*strongly agree*) with the middle response alternative labeled *undecided*.

#### Vaccine recommendation behaviors

Respondents were presented with three questions about how frequently they recommend COVID-19 vaccines. More specifically, the questions concerned what proportion of their unvaccinated adult, adolescent, or pregnant patients they recommend COVID-19 vaccines to. The response alternatives ranged from 0% (*I do not actively recommend it to any of these patients*) to 100% (*I actively recommend it to all of these patients*) with 10-unit intervals and the additional alternative *I do not treat patients within this age/target group*. If a physician chose the last alternative, we administered a question querying their intentions to recommend the vaccine if they would treat such patients (response scale: 0% [*I would never recommend it*] to 100% [*I would certainly recommend it*] with 10-unit intervals) and included their response to this question in the vaccine-recommendation behavior variable instead.

### Statistical analysis

The statistical analysis plan was pre-registered prior to data analysis but after data collection (as explained at: https://osf.io/jzn2k). We compared the physicians’ attitudes to mandates between countries in three one-way ANOVAs-one for each mandate attitude item. Each mandate attitude item was included as the dependent variable and country as an independent variable. Significant results were followed up with post-hoc Tukey’s tests, where all potential pair-wise comparisons between country means are conducted while controlling for multiple tests.

Linear regression analyses were conducted separately for each country to investigate the relationship between vaccination attitudes and attitudes to mandates within each country context. The responses to the vaccination attitude questions were averaged within constructs (reliability coefficients of the constructs were between .57 and .93; see Table S3). Each construct was thus represented by a single variable. The mandate attitude variables were included as outcomes in separate analyses, each with the 11 vaccination-attitude constructs as predictors. Gender and age were included as control variables. As age was recorded as a categorical variable (<40 years old; 40–49 years old; 50 years or older), the variable was contrast coded so that each category was compared to the preceding category. The categorization was conducted to increase respondent anonymity and the cutoffs were chosen on an arbitrary basis.

We investigated the relationship between mandate attitudes and recommendation behaviors in each country with Spearman’s rank correlations. For this, the three mandate variables were merged into one variable representing the physicians’ average attitude to mandates (Cronbach’s alpha = .76), except in Germany, where only the two items administered were used (Spearman-Brown reliability for two items = .78). Also the three recommendation behavior variables were averaged across items (Cronbach’s alpha = .68).

Due to the large sample size and multiple tests, only *p*-values < .001 were considered statistically significant. Analyses were conducted in R version 4.2.2^38^ The R package rstatix version 0.7.1^39^ was used for ANOVAs and follow-up analyses.

## Results

### Sample characteristics

The survey was filled out by *n* = 389 physicians in Finland, *n* = 1299 in France, *n* = 607 in Germany, and *n* = 580 in Portugal. We only included individuals with no missing data on all measures included in the present study. Because of this, one respondent was excluded from the Finnish sample (who reported having provided an incorrect response to a question by mistake and the response was coded as missing; final Finnish sample *n* = 388), and 78 respondents from the French sample (the survey in France allowed respondents to skip questions; final French sample *n* = 1221). No other exclusion criteria were applied. The total sample of physicians (Table 2) was thus *N* = 2796. Posthoc power analyses for each country sample can be found in the Supplementary Material.

**Table 2.** Descriptive information about the sample of physicians.

Characteristic	Finland (*n* = 388)		France (*n* = 1221)		Germany (*n* = 607)		Portugal (*n* = 580)		Total (*N* = 2796)	
	*n*	%	*n*	%	*n*	%	*n*	%	*n*	%
Gender										
Male	86	22.16	542	44.39	375	61.78	117	20.17	1120	40.06
Female	302	77.84	675	55.28	228	37.56	463	79.83	1668	59.66
Non–binary	–	–	–	–	2	0.33	–	–	2	0.07
Prefer not to state	–	–	4	0.33	2	0.33	–	–	6	0.21
Age										
<40	55	14.18	397	32.51	86	14.17	387	66.72	925	33.08
40–49	81	20.88	326	26.70	116	19.11	113	19.48	636	22.75
50–	252	64.95	498	40.79	405	66.72	80	13.79	1235	44.17
Profession										
GP	262	67.53	1215	99.51	412	67.87	245	42.24	2134	76.32
Pediatrician	112	28.87	3	0.25	80	13.18	312	53.79	507	18.13
Gynecologist	–	–	2	0.16	115	18.95	–	–	117	4.18
Other	14	3.61	1	0.08	–	–	23	3.97	38	1.36
Work tasks										
Involve vaccination	362	93.30	1220	99.92	603	99.34	573	98.79	2758	98.64
Does not involve vaccination	26	6.70	1	0.08	4	0.66	7	1.21	38	1.36
COVID-19 vaccinated										
No	1	0.26	3	0.25	11	1.81	5	0.86	20	0.72
Partially	0	0.00	5	0.41	4	0.66	1	0.17	10	0.36
Fully	15	3.87	48	3.39	34	5.60	38	6.55	135	4.83
Boosted	372	95.88	1165	95.41	558	91.93	536	92.41	2631	94.10

The response alternative to the question of whether the HCP was vaccinated against COVID-19 included descriptions of what partially, fully, or boosted meant in the relevant country at the time of data collection. For example: “No;” “Yes, I am partially vaccinated (one dose of the Pfizer-BioNTech, Moderna, or Oxford/AstraZeneca vaccine);” “Yes, I am fully vaccinated (two doses of the Pfizer-BioNTech, Moderna, or Oxford/AstraZeneca vaccine, or one dose of the Johnson & Johnson vaccine); “Yes, I am fully vaccinated and received a booster (third dose of Pfizer-BioNTech or Moderna, or a second dose of the Johnson & Johnson vaccine).”

The Finnish, French, and Portuguese samples consisted of a larger proportion of women than men, whereas the majority of the German respondents were men. Only eight individuals in the total sample reported their gender as other than male or female. Due to the low number, these individuals were excluded from the regression analyses. In Finland, France, and Germany, the oldest age category (50 years or older) was the largest, whereas most Portuguese respondents were in the youngest age category (<40 years old). GPs were the most common professional group in all countries except for Portugal, where the majority were pediatricians. Almost all physicians reported that their work tasks involved vaccination and that they had been vaccinated against COVID-19.

### Agreement with COVID-19 mandates

The response distributions of all items are reported in Table S4. Most physicians (78%) agreed (i.e., chose the response alternatives 4 or 5 on the 5-point scale) with the statement that COVID-19 vaccines should be mandatory for HCPs, whereas about half agreed with the statement that COVID-19 vaccine should be mandatory for the public (49%). A majority also agreed with the use of a COVID-19 health pass (67%).

### Between-country comparisons of attitudes to mandates

**Figure 1.**
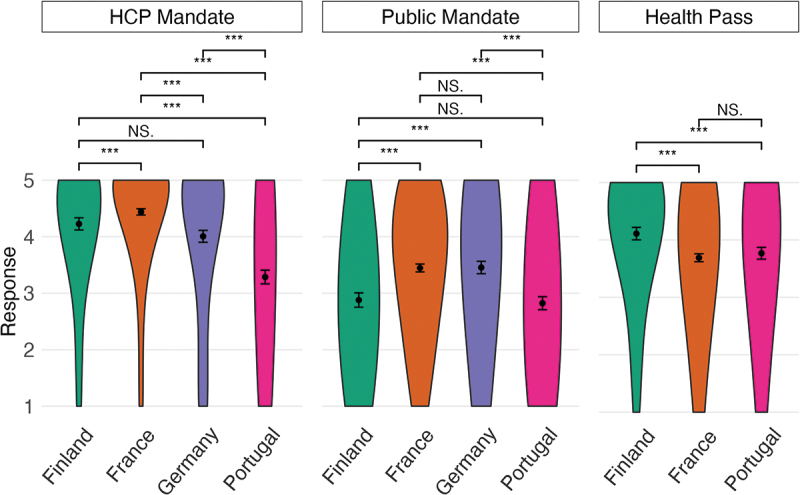
Comparisons of the physicians’ attitudes to COVID-19 vaccine mandates between countries.

Figure 1 shows the physicians’ attitudes to COVID-19 vaccine mandates by country (means and standard deviations are reported in Table S5). All three one-way ANOVAs testing if the physicians’ attitudes to the mandates differed between countries were statistically significant (HCP mandate; *F*[3, 2792] = 124.61, *p* < .001; public mandate: *F*[3, 2792] = 44.63, *p* < .001; health pass: *F*[2, 2186] = 17.64, *p* < .001). Follow-up, pair-wise comparisons (Table S6) revealed that the physicians in France reported the strongest agreement with a mandate for HCPs; significantly stronger than those in Finland, Germany, and Portugal. The physicians in Portugal reported significantly lower agreement with HCP mandates than those in all other countries. French and German physicians reported significantly stronger agreement with public vaccine mandates than Finnish and Portuguese physicians. Lastly, physicians in Finland reported significantly stronger agreement with the use of a health pass than physicians in France and Portugal.

HCPs’ agreement with COVID-19 vaccine mandates for HCPs, COVID-19 vaccine mandates for the public, and the use of a COVID-19 health pass on a scale from 1 (strongly disagree) to 5 (strongly agree). The outer borders of the violin shapes represent the distribution of responses within a country. Dots represent means and bars 95% CIs. The question about a health pass was not administered in Germany.

### Predictors of attitudes to COVID-19 vaccine mandates by country

**Figure 2.**
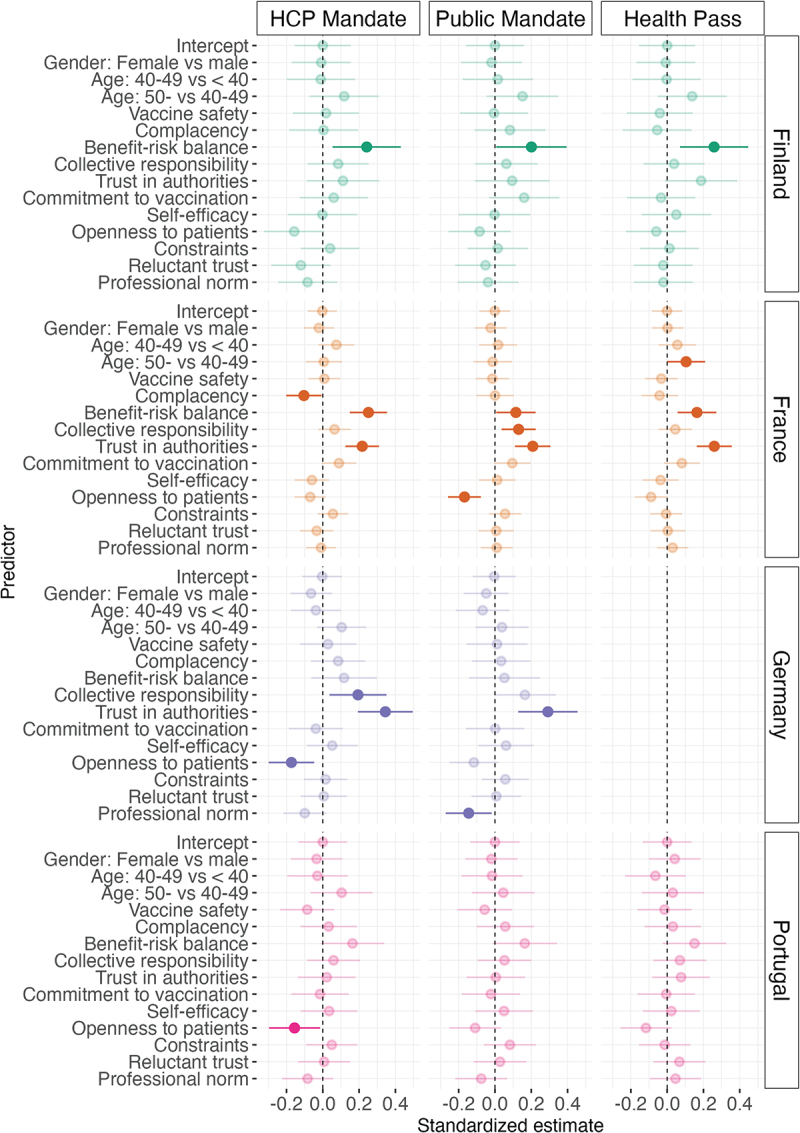
Predictors of the physicians’ attitudes to COVID-19 vaccine mandates by country.

The results from the linear regression analyses investigating vaccination attitudes as predictors of attitudes to COVID-19 mandates in each country are presented in Figure 2 and Table S7. The control variables gender and age did not significantly predict mandate attitudes in any of the countries, except that in France, 40–49-year-old physicians were significantly more positive toward health passes than younger physicians. For the main predictors of interest, the pattern of variables predicting mandate attitudes differed between countries and between mandate types, and most relationships were small to neglectable. In the following sections, statistically significant results are reported by country.

Results from the linear regression analyses on predictors of attitudes to mandates by country. Dots represent standardized beta coefficients and bars 99.9% CIs. Transparent dots represent non-significant coefficients whereas saturated dots are statistically significant at *p* < .001. Standardized beta coefficients, standard errors, and *p*-values can be found in Table S7.

In Finland, larger perceived vaccine benefits compared to risks were significantly related to more positive attitudes toward all three types of mandates. In France, larger perceived vaccine benefits compared to risks and greater trust in authorities were significantly related to more positive attitudes to all mandate types. In addition, lower complacency was significantly related to more positive attitudes to COVID-19 vaccines being mandated for HCPs, and larger perceived collective responsibility and lower openness to patients were significantly related to more positive attitudes to COVID-19 vaccines being mandated for the public. In Germany, greater trust in authorities was significantly related to more positive attitudes toward both mandate types measured. Furthermore, higher perceived collective responsibility and less openness to patients were significantly associated with more positive attitudes to COVID-19 vaccine mandates for HCPs, whereas perceiving the professional norm to be less in favor of vaccines was significantly related to more positive attitudes toward COVDI-19 vaccine mandates for the public. In Portugal, the only statistically significant result was that less openness to patients was significantly associated with more positive attitudes to COVID-19 vaccine mandates for HCPs.

### Relationship between mandate attitudes and vaccine recommendation behaviors

**Figure 3.**
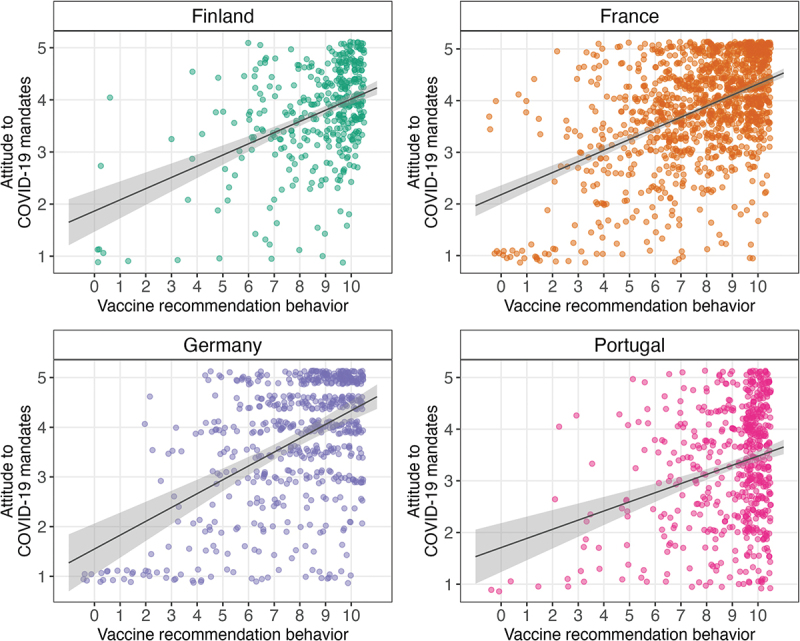
Correlation between COVID-19 vaccine mandate attitudes and vaccine recommendation behavior by country.

The response distribution of the recommendation behavior variable in each country is shown in Figure S1. There was a statistically significant relationship between HCPs’ attitudes to COVID-19 mandates and their vaccine recommendation behavior in all countries, suggesting that HCPs with more positive mandate attitudes recommended COVID-19 vaccines more frequently (Figure 3). The relationships were moderate in Germany (*r*_*s*_ = .39, *p* < .001), France (*r*_*s*_ = .39, *p* < .001), and Finland (*r*_*s*_ = .38, *p* < .001), but weak in Portugal (*r*_*s*_ = .21, *p* < .001).

Correlations between attitudes to COVID-19 mandates (higher number indicates more positive attitudes) and vaccine recommendation behavior (higher number indicates more frequent vaccine recommendation) in each country and 99.9% CIs. Dots have been jittered to facilitate interpretation.

## Discussion

In the present study, we investigated the degree to which physicians in four European countries supported COVID-19 vaccine mandates, as well as whether the level of support differed between countries and was related to vaccination attitudes and vaccine recommendation behaviors. Across all countries, a clear majority of the physicians (78%) were in favor of COVID-19 vaccine mandates for HCPs, whereas opinions were more divided when it came to COVID-19 vaccine mandates for the public (49% agreed). A majority also favored the use of a COVID-19 health pass (67%).

The higher support for HCP mandates compared to public mandates is in line with previous research on mandates related to COVID-19 vaccines.^19^ We also found significant differences among the four countries in how much physicians agreed with the mandates. These differences in mandate attitudes could be a result of socio-political differences between countries. In the present study, mandate acceptance was higher in countries where the mandates in question were in effect than in countries where they were not. For instance, physicians were more in favor of COVID-19 mandates for HCPs in Finland, France, and Germany, where such mandates had been implemented than in Portugal, where no such mandates were in place. Furthermore, none of the countries had implemented COVID-19 vaccine mandates for the public, and, in line with this, physicians had more negative attitudes toward public mandates than HCP mandates. However, physicians were more positive toward COVID-19 vaccine mandates for the public in countries where public mandates for other vaccines were in place (i.e., France and Germany) than in countries without public mandates (i.e., Finland and Portugal). This is in line with previous qualitative data that suggests an association between existing vaccine mandates and support for COVID-19 vaccine mandates.^40^ It is possible that experience with mandates increases support for mandates (e.g., as a result of becoming used to them) or that mandates have been implemented in populations more receptive to them to begin with (e.g., because it aligns with other policies or the way healthcare is implemented in the country). The latter possibility seems less plausible as mandates tend to be introduced when vaccine uptake is low and hesitancy toward vaccines is more prevalent.^23,41^

The relationships between vaccination attitudes and mandate attitudes were mostly small to zero. The most consistent results were that higher perceived vaccine benefits compared to risks and higher trust in authorities were related to more positive mandate attitudes. This, however, differed among countries. For example, mandate attitudes were more related to the benefits and risks of vaccination in Finland, whereas in Germany, they were more related to trust in health authorities. Also, more positive attitudes to mandates were related to more frequent COVID-19 vaccine recommendation behavior. These results are in line with previous studies on HCPs, which have found similar vaccine-attitude constructs to be related to support for vaccine mandates.^17,22^ Additional related factors identified in research on other vaccines are perceived collective responsibility and complacency.^22^ These factors were significant predictors in some of our analyses, but the relationships were small and non-systematic.

Research indicates that vaccine mandates for HCPs and the public, as well as health passes, have had positive effects on vaccine uptake and public health.^5,42–46^ However, vaccine mandates are a complex issue, and the evaluation preceding mandate implementation should be situation-specific and consider a broad range of factors, such as the risks of the disease, the safety and effectiveness of the vaccine, the availability of other protective measures that are less coercive, and the potential consequences of the mandates.^9,13^ The present study contributes to the body of research with two primary conclusions. First, physicians’ attitudes to mandates were more positive in countries and for target groups already subject to COVID-19 or other vaccine mandates. This suggests that implementing mandates in settings without a history of similar mandates might require extra caution. Second, attitudes to mandates were mostly unrelated to physicians’ vaccination attitudes, which suggests that negative mandate attitudes might be rooted in other factors than concerns about vaccinations, for example, political ideology or ethical convictions.^47^ Nevertheless, it is also possible lower variance in physicians’ vaccination attitudes (e.g., compared to the general public) made it difficult to detect relationships with their mandate attitudes.

When introducing mandates, it is important to keep in mind that mandating vaccination might cause resistance from the public^48,49^ and forcing vaccine-reluctant individuals to vaccinate can result in nocebo effects from the vaccines.^50,51^ A common concern is that implementing mandates leads to decreased trust in authorities, which, in turn, can have several negative consequences.^10^ However, low vaccine uptake in the absence of mandates might also increase mistrust in authorities by increasing disease risk, mortality, and fear.^52^ If implemented, mandates should not be the only intervention authorities apply to increase vaccine uptake and vaccine mandates should always be accompanied by information about vaccines in well-targeted, action-oriented, and evidence-based communication campaigns. Vaccine communication and education may even be more important when vaccines are mandated.^53^

### Limitations

A limitation of the present study is the potential for nonresponse bias. Demographic characteristics of HCPs answering online surveys have been found to significantly differ from those who do not respond, for example with respect to age and workload.^54^ To reduce the risk of nonresponse bias, the survey was carefully designed to be relevant and user-friendly (e.g., through cultural adaptation, cognitive interviewing, and piloting), study participation was completely anonymous, and reminders were sent out when possible. Also, the fact that all respondents in the present study were physicians, and that mandate questions concerned COVID-19 vaccines only, may limit generalizability to other HCPs and vaccines. Furthermore, in France, we recruited physicians who have medical students in their practices. These physicians may report attitudes closer to the official recommendations than their colleagues without students.

Another limitation is the fact that there were demographic differences between the samples, which could explain the differences in mandate attitudes between countries. However, the demographic variables were not significantly related to mandate attitudes within the countries (except for a small, significant difference between the oldest and the younger age groups in France), suggesting that between-country differences were not driven by demographic differences between samples.

Lastly, the small number of countries included in the data collection limits the possibility to conduct generalizable statistical analyses and inferences on the relationship between the mandate situation in a country and attitudes to mandates. The observation that mandate attitudes were more positive when mandates were in effect in the respective countries should be replicated in a larger sample of countries.

## Conclusion

The results of the present study showed that physicians in Finland, France, Germany, and Portugal were mostly positive toward COVID-19 vaccines being mandatory for HCPs, whereas attitudes to COVID-19 vaccine mandates for the public were divided. A majority agreed with the implementation of a health pass. Mandate attitudes varied between countries, with physicians reporting more positive attitudes to COVID-19 mandates in countries that had already implemented those mandates. Although physicians with more positive mandate attitudes perceived vaccines as more beneficial (in Finland and France) and had greater trust in medical authorities (in France and Germany), most vaccination-attitude constructs were very weakly related to attitudes to COVID-19 mandates. In addition to replicating the results of the present study, future research should investigate socio-political factors that could explain differences between countries in mandate attitudes. The present research provides information that supports the evaluation of mandate implementation when it comes to existing vaccines as well as potential vaccines developed during future pandemics.

## Supplementary Material

Supplemental Material

## References

[1] Patel MK, Orenstein WA. Classification of global measles cases in 2013–17 as due to policy or vaccination failure: a retrospective review of global surveillance data. Lancet Glob Health. 2019;7(3):e313–10. doi:10.1016/S2214-109X(18)30492-3. 30784632

[2] Fine P, Eames K, Heymann DL. “Herd immunity”: a rough guide. Clin Infect Dis. 2011;52(7):911–6. doi:10.1093/cid/cir007. 21427399

[3] Metcalf CJE, Ferrari M, Graham AL, Grenfell BT. Understanding herd immunity. Trends Immunol. 2015;36(12):753–5. doi:10.1016/j.it.2015.10.004. 26683689

[4] Gravagna K, Becker A, Valeris-Chacin R, Mohammed I, Tambe S, Awan FA, Toomey TL, Basta NE. Global assessment of national mandatory vaccination policies and consequences of non-compliance. Vaccine. 2020;38(49):7865–73. doi:10.1016/j.vaccine.2020.09.063. 33164808 PMC8562319

[5] Vaz OM, Ellingson MK, Weiss P, Jenness SM, Bardají A, Bednarczyk RA, Omer SB. Mandatory vaccination in Europe. Pediatrics. 2020;145(2). doi:10.1542/peds.2019-0620. PMC7011108 31932361

[6] Odone A, Dallagiacoma G, Frascella B, Signorelli C, Leask J. Current understandings of the impact of mandatory vaccination laws in Europe. Expert Rev Vaccines. 2021;20(5):559–75. doi:10.1080/14760584.2021.1912603. 33896302

[7] Vergallo GM, Del Rio A, Negro F, Zaami S. COVID-19 vaccine mandates: what are the current European public perspectives? Eur Rev Med Pharmacol Sci. 2022;26(2):643–52. doi:10.26355/eurrev_202201_27891. 35113440

[8] Burki T. COVID-19 vaccine mandates in Europe. Lancet Infect Dis. 2022;22(1):27–8. doi:10.1016/S1473-3099(21)00776-3. 34953554 PMC8694743

[9] Giubilini A, Savulescu J, Pugh J, Wilkinson D. Vaccine mandates for healthcare workers beyond COVID-19. J Med Ethics. 2022;22(3):211–20. doi:10.1136/medethics-2022-108229. PMC9985724 35636917

[10] Maneze D, Salamonson Y, Grollman M, Montayre J, Ramjan L. Mandatory COVID-19 vaccination for healthcare workers: a systematic review. Int J Nurs Stud. 2023;138:104389. doi:10.1016/j.ijnurstu.2022.104389. 36462385 PMC9709452

[11] Maltezou HC, Botelho-Nevers E, Brantsæter AB, Carlsson RM, Heininger U, Hübschen JM, Josefsdottir KS, Kassianos G, Kyncl J, Ledda C, et al. Vaccination of healthcare personnel in Europe: update to current policies. Vaccine. 2019;37(52):7576–84. doi:10.1016/j.vaccine.2019.09.061. 31623916

[12] MacDonald NE, Harmon S, Dube E, Steenbeek A, Crowcroft N, Opel DJ, Faour D, Leask J, Butler R. Mandatory infant & childhood immunization: rationales, issues and knowledge gaps. Vaccine. 2018;36(39):5811–8. doi:10.1016/j.vaccine.2018.08.042. 30143274

[13] Omer SB, Betsch C, Leask J. Mandate vaccination with care. Nature. 2019;571(7766):469–72. doi:10.1038/d41586-019-02232-0. 31332351

[14] Shachar C, Reiss DR. When are vaccine mandates appropriate? AMA J Ethics. 2020;22:36–42. doi:10.1001/amajethics.2020.36. 31958389

[15] Charron J, Gautier A, Jestin C. Influence of information sources on vaccine hesitancy and practices. Med Mal Infect. 2020;50(8):727–33. doi:10.1016/j.medmal.2020.01.010. 32067795

[16] Eller NM, Henrikson NB, Opel DJ. Vaccine information sources and parental trust in their child’s health care provider. Health Edu Behav. 2019;46(3):445–53. doi:10.1177/1090198118819716. PMC7872219 30616381

[17] Giannakou K, Kyprianidou M, Christofi M, Kalatzis A, Fakonti G. Mandatory COVID-19 vaccination for healthcare professionals and its association with general vaccination knowledge: a nationwide cross-sectional survey in cyprus. Front Public Health. 2022;10. doi:10.3389/fpubh.2022.897526. PMC9130732 35646772

[18] Shaw J, Hanley S, Stewart T, Salmon DA, Ortiz C, Trief PM, Asiago Reddy E, Morley CP, Thomas SJ, Anderson KB. Healthcare personnel (HCP) attitudes about coronavirus disease 2019 (COVID-19) vaccination after emergency use authorization. Clin Infect Dis. 2022;75(1):e814–e21. doi:10.1093/cid/ciab731. 34467370 PMC8513411

[19] Mayan D, Nguyen K, Keisler B, Rowley JA. National attitudes of medical students towards mandating the COVID-19 vaccine and its association with knowledge of the vaccine. PloS One. 2021;16(12):e0260898. doi:10.1371/journal.pone.0260898. 34936665 PMC8694456

[20] Jain J, Saurabh S, Kumar P, Verma MK, Goel AD, Gupta MK, Bhardwaj P, Raghav PR. COVID-19 vaccine hesitancy among medical students in India. Epidemiol Infect. 2021;149:1–0. doi:10.1017/S0950268821001205. PMC8185413 34011421

[21] Gualano MR, Corradi A, Voglino G, Catozzi D, Olivero E, Corezzi M, Bert F, Siliquini R. Healthcare workers’ (HCWs) attitudes towards mandatory influenza vaccination: a systematic review and meta-analysis. Vaccine. 2021;39(6):901–14. doi:10.1016/j.vaccine.2020.12.061. 33451776

[22] Verger P, Botelho-Nevers E, Garrison A, Gagnon D, Gagneur A, Gagneux-Brunon A, Dubé E. Vaccine hesitancy in health-care providers in Western countries: a narrative review. Expert Rev Vaccines. 2022;21(7):909–27. doi:10.1080/14760584.2022.2056026. 35315308

[23] Verger P, Dualé C, Scronias D, Lenzi N, Pulcini C, Launay O. Attitudes of hospital physicians toward childhood mandatory vaccines in France: a cross-sectional survey. Hum Vaccin Immunother. 2022;18(1). doi:10.1080/21645515.2020.1870393. PMC8920152 33616464

[24] THL. Sosiaali- ja terveysalan henkilöstön rokotukset [Internet]; 2023 [accessed 2023 May 4]. https://thl.fi/fi/web/infektiotaudit-ja-rokotukset/tietoa-rokotuksista/eri-kohderyhmien-rokottaminen/sosiaali-ja-terveysalan-henkiloston-rokotukset.

[25] Ward JK, Colgrove J, Verger P. Why France is making eight new vaccines mandatory. Vaccine. 2018;36(14):1801–3. doi:10.1016/j.vaccine.2018.02.095. 29506923

[26] Bundesministerium für Gesundheit. Masernschutzgesetz [Internet]; 2023 [accessed 2023 May 4]. https://www.masernschutz.de/.

[27] Fasce A, Karlsson L, Verger P, Mäki O, Taubert F, Garrison A, Schmid P, Holford DL, Lewandowsky S, Rodriguesa F, et al. Endorsement of alternative medicine and vaccine hesitancy among physicians: a cross-sectional study in four European countries. Hum Vaccin Immunother. 2023;19(2). doi:10.1080/21645515.2023.2242748. PMC10431744 37581343

[28] Holford DL, Schmid P, Fasce A, Garrison A, Karlsson LC, Taubert F, Verger P, Lewandowsky S, Fisher H, Betsch C, et al. Difficulties faced by physicians from four European countries in rebutting anti-vaccination arguments. PsyArxiv [Internet]; 2023 [accessed 2023 Aug 15]. https://psyarxiv.com/j3526/.

[29] Peterson CH, Peterson NA, Powell KG. Cognitive interviewing for item development: validity evidence based on content and response processes. Meas Eval Couns Dev. 2017;50(4):217–23. doi:10.1080/07481756.2017.1339564.

[30] Garrison A, Karlsson L, Fressard L, Fasce A, Rodrigues F, Schmid P, Taubert F, Holford D, Lewandowsky S, Nynäs P, et al. International adaptation and validation of the Pro-VC-Be: measuring the psychosocial determinants of vaccine confidence in healthcare professionals in European countries. Expert Rev Vaccines. 2023;22(1):726–37. doi:10.1080/14760584.2023.2242479. 37507356

[31] Verger P, Fressard L, Soveri A, Dauby N, Fasce A, Karlsson L, Lewandowsky S, Schmid P, Dubé E, Gagneur A. An instrument to measure psychosocial determinants of health care professionals’ vaccination behavior: validation of the Pro-VC-Be questionnaire. Expert Rev Vaccines. 2022;21(5):693–709. doi:10.1080/14760584.2022.2046467. 35238274

[32] Garrison A, Fressard L, Karlsson L, Soveri A, Fasce A, Lewandowsky S, Schmid P, Gagneur A, Dubé E, Verger P. Measuring psychosocial determinants of vaccination behavior in healthcare professionals: validation of the Pro-VC-Be short-form questionnaire. Expert Rev Vaccines. 2022;21(10):1505–14. doi:10.1080/14760584.2022.2108800. 35938710

[33] Michie S, Johnston M, Abraham C, Lawton R, Parker D, Walker A. Making psychological theory useful for implementing evidence based practice: a consensus approach. Qual Saf Health Care. 2005;14(1):26–33. doi:10.1136/qshc.2004.011155. 15692000 PMC1743963

[34] McSherry LA, Dombrowski SU, Francis JJ, Murphy J, Martin CM, O’Leary JJ, Sharp L. ‘It’s a can of worms’: understanding primary care practitioners’ behaviours in relation to HPV using the theoretical domains framework. Implement Sci. 2012;7(1). doi:10.1186/1748-5908-7-73. PMC3523072 22862968

[35] Rosenstock IM. Why people use health services. Milbank Mem Fund Q. 1966;44(3):94–124. doi:10.2307/3348967. 5967464

[36] Carpenter CJ. A meta-analysis of the effectiveness of health belief model variables in predicting behavior. Health Commun. 2010;25(8):661–9. doi:10.1080/10410236.2010.521906. 21153982

[37] Betsch C, Schmid P, Heinemeier D, Korn L, Holtmann C, Böhm R, Angelillo IF. Beyond confidence: development of a measure assessing the 5C psychological antecedents of vaccination. PloS One. 2018;13(12):1–32. doi:10.1371/journal.pone.0208601. PMC6285469 30532274

[38] R Core Team. R: a language and environment for statistical computing [Internet]; 2022. https://www.R-project.org/.

[39] Kassambara A. Rstatix: pipe-friendly framework for basic statistical tests [Internet]; 2022. https://CRAN.R-project.org/package=rstatix.

[40] Attwell K, Rizzi M, McKenzie L, Carlson SJ, Roberts L, Tomkinson S, Blyth CC. COVID-19 vaccine mandates: an Australian attitudinal study. Vaccine. 2022;40(51):7360–9. doi:10.1016/j.vaccine.2021.11.056. 34872796 PMC8629747

[41] Attwell K, Navin MC, Lopalco PL, Jestin C, Reiter S, Omer SB. Recent vaccine mandates in the United States, Europe and Australia: a comparative study. Vaccine. 2018;36(48):7377–84. doi:10.1016/j.vaccine.2018.10.019. 30337171

[42] Lytras T, Kopsachilis F, Mouratidou E, Papamichail D, Bonovas S. Interventions to increase seasonal influenza vaccine coverage in healthcare workers: a systematic review and meta-regression analysis. Hum Vaccin Immunother. 2016;12(3):671–81. doi:10.1080/21645515.2015.1106656. 26619125 PMC4964628

[43] Carrera M, Lawler EC, White C. Population mortality and laws encouraging influenza vaccination for hospital workers. Ann Intern Med. 2021;174(4):444–52. doi:10.7326/M20-0413. 33395343

[44] Howard-Williams M, Soelaeman RH, Fischer LS, McCord R, Davison R, Dunphy C. Association between state-issued COVID-19 vaccine mandates and vaccine administration rates in 12 US states and the District of Columbia. JAMA Health Forum. 2022;3(10):e223810. doi:10.1001/jamahealthforum.2022.3810. 36306119 PMC9617176

[45] McGarry BE, Gandhi AD, Syme M, Berry SD, White EM, Grabowski DC. Association of state COVID-19 vaccine mandates with staff vaccination coverage and staffing shortages in US nursing homes. JAMA Health Forum. 2022;3(7):e222363. doi:10.1001/jamahealthforum.2022.2363. 35983581 PMC9338409

[46] Karaivanov A, Kim D, Lu SE, Shigeoka H. COVID-19 vaccination mandates and vaccine uptake. Nat Hum Behav. 2022;6(12):1615–24. doi:10.1038/s41562-022-01363-1. 35654962

[47] Smith LE, Hodson A, Rubin GJ. Parental attitudes towards mandatory vaccination; a systematic review. Vaccine. 2021;39(30):4046–53. doi:10.1016/j.vaccine.2021.06.018. 34140173

[48] Sprengholz P, Felgendreff L, Böhm R, Betsch C. Vaccination policy reactance: predictors, consequences, and countermeasures. J Health Psychol. 2022;27(6):1394–407. doi:10.1177/13591053211044535. 34488460 PMC9036150

[49] Betsch C, Böhm R. Detrimental effects of introducing partial compulsory vaccination: experimental evidence. Eur J Public Health. 2016;26(3):378–81. doi:10.1093/eurpub/ckv154. 26297722

[50] Ward J, Gauna F, Gagneux-Brunon A, Botelho-Nevers E, Cracowski J-L, Khouri C, Launay O, Verger P, Peretti-Watel P. The French health pass holds lessons for mandatory COVID-19 vaccination. Nat Med. 2022;28(2):232–5. doi:10.1038/s41591-021-01661-7. 35022575

[51] Khouri C, Larabi A, Verger P, Gauna F, Cracowski J-L, Ward J. Impact of vaccine hesitancy on onset, severity and type of self-reported adverse events: a French cross-sectional survey. Drug Saf. 2022;45(10):1049–56. doi:10.1007/s40264-022-01220-0. 35972651 PMC9379877

[52] Lewandowsky S, Holford D, Schmid P. Public policy and conspiracies: the case of mandates. Curr Opin Psychol. 2022;47:101427. doi:10.1016/j.copsyc.2022.101427. 36029701 PMC9296372

[53] Attwell K, Ward JK, Tomkinson S. Manufacturing consent for vaccine mandates: a comparative case study of communication campaigns in France and Australia. Front Commun. 2021;6. doi:10.3389/fcomm.2021.598602.

[54] Verger P, Scronias D, Fradier Y, Meziani M, Ventelou B. Online study of health professionals about their vaccination attitudes and behavior in the COVID-19 era: addressing participation bias. Hum Vaccin Immunother. 2021;17(9):2934–9. doi:10.1080/21645515.2021.1921523. 34047670 PMC8381780

