# Supplementary material for: Healthcare professionals’ attitudes to mandatory COVID-19 vaccination: Cross-sectional survey data from four European countries
Source: Hum Vaccin Immunother. 2023 Sep 19;19(2):2256442. doi: 10.1080/21645515.2023.2256442 (PMC10512846; doi:10.1080/21645515.2023.2256442)

Supplementary Material

Table S1

Information about Recruitment Procedures

Country	Physician type targeted	Recruitment channel	Data collection period	Number of invitations sent	Number of reminders sent	Incentives	Pre-registration
Finland	GPs, pediatricians	Email lists of The Finnish Medical Association[a]	April 25th–May 16th	2,828	2	no	https://osf.io/586jb
France	GPs	Email lists of the National College of Teaching Generalists	March 18th–April 27th	3,499	4	no	no
Germany	GPs, pediatricians, gynecologists	The panel provider Schlesinger Group Germany	March 31st–April 24th	NA	NA	incentivized by the panel provider (amount unknown)	https://aspredicted.org/CTZ_N1B
Portugal	GPs, pediatricians	Email lists of the Portuguese Society of Pediatricians and the Portuguese Association of General Practice and Family Medicine	April 6th–May 6th	~5,500	0	10€ shopping voucher	https://osf.io/j7as2

Note. GP = General practitioner. In Finland and Germany, mechanisms to prevent multiple entries were available. [a]Includes almost all medical doctors in Finland. The survey was sent to GPs and pediatricians who were members of the Finnish Medical Association, who had an active medical license, lived in Finland, and were 70 years or younger.

Table S2

Survey Questions

Construct	Statement	Label
Attitudes to mandates[a]	Vaccination against Covid-19 should be mandatory for healthcare workers in my country	mand_hcp
	Vaccination against Covid-19 should be mandatory for the general public in my country	mand_public
	I agree with the use of a 'health pass' to allow vaccinated individuals to access public spaces and to travel[b]	mand_pass
Perceived vaccine risks[a]	Vaccines against measles are safe	safe_measles
	Vaccines against influenza are safe	safe_flu
	Vaccines against hepatitis B are safe	safe_hepb
	Vaccines against human papillomaviruses are safe	safe_hpv
Complacency[a]	Today, some vaccines recommended by [relevant authority] are not useful, because the diseases they prevent are not serious	comp_useful
	Children are vaccinated against too many diseases	comp_many
	Children are vaccinated at too young an age	comp_young
Perceived benefit-risk balance[a,c]	The benefits of the vaccine against measles outweigh its potential risks	balance_measles
	The benefits of the vaccine against influenza outweigh its potential risks	balance_flu
	The benefits of the vaccine against hepatitis B outweigh its potential risks	balance_hepb
	The benefits of the vaccine against human papillomaviruses outweigh its potential risks	balance_hpv
	The benefits of the vaccines against Covid-19 available in my country outweigh their potential risks	balance_cov
Perceived collective responsibility[a]	I recommend the vaccines on the vaccination schedule to my patients because it's essential to contribute to the protection of the population (community immunity)	coll_essential
	I recommend the vaccines in the official schedule to my hesitant patients, explaining to them the importance of community immunity	coll_explain
Trust in authorities[a]	I trust the information provided by the [relevant authority] about the risks and benefits of vaccines	trust_info
	I trust the [relevant authority] to establish the vaccination strategy	trust_strategy
	I trust the [relevant authority] to ensure that vaccines are safe	trust_safe
Commitment to vaccination[a]	I am committed in ensuring that my patients are vaccinated.	commit_vax
	I am committed to keeping my knowledge about vaccination up-to-date (e.g., through CME, conferences, reading)	commit_knowledge
	I am committed to developing the skills needed to communicate better with my patients about vaccination	commit_communic
Self-efficacy[a]	I feel comfortable advising my patients about the risks and benefits of vaccines	effic_advice
	I feel comfortable discussing vaccines with my patients who are highly hesitant about vaccination	effic_discuss
	I feel sufficiently trained and informed to discuss vaccines with all patients	effic_traindisc
	I feel sufficiently trained on how to bring up the question of vaccines with hesitant patients	effic_trainbring
Openness to patients[a]	Patients who are hesitant about the benefits and risks of vaccines have legitimate questions	open_legit
	I inform my patients about the benefits and risks of vaccines without trying to influence them	open_influence
	I am open to patients delaying immunization of their children	open_delay

Perceived constraints[a]	The cost of some vaccines is a problem for some patients and can keep me from prescribing them	const_cost
	The lack of availability of certain vaccines in my country is sometimes a problem that can keep me from prescribing them to my patients	const_country
	The lack of availability of certain vaccines in my place of work is sometimes a problem that can keep me from prescribing them to my patients	const_practice
Reluctant trust[a]	I may sometimes recommend vaccines from the official schedule even if I feel I am not sufficiently informed	reluct_inform
	I may sometimes recommend vaccines from the official schedule even if I feel the vaccination policy is not sufficiently clear	reluct_policy
	I may sometimes recommend the vaccines on the official schedule even in cases where I have doubts about their safety	reluct_safety
Perceived professional norms[a]	I think that most medical doctors in my country recommend that people get vaccinated	norm_recom
	I think that most medical doctors in my country are in favour of vaccination	norm_favour
Recommendation behavior	When you treat adults who have not had the Covid-19 vaccine, what is the percentage of these patients for whom you actively recommend the vaccine?	
	(administered if HCP does not treat adults: Please imagine you are treating an adult who has not had the Covid-19 vaccine and has no contraindications. How likely is it that you would recommend the vaccine to the patient?)	
	When you treat [age range] old adolescents who have not had the Covid-19 vaccine, what is the percentage of these patients for whom you actively recommend the vaccine?	
	(administered if HCP does not treat adolescents: Please imagine you are treating a [age range] old adolescent who has not had the Covid-19 vaccine and has no contraindications. How likely is it that you would recommend the vaccine to the patient?)	
	When you treat pregnant women who have not had the Covid-19 vaccine, what is the percentage of these patients for whom you actively recommend the vaccine?	
	(administered if HCP does not treat pregnant women: Please imagine you are treating a pregnant woman who has not had the Covid-19 vaccine and has no contraindications. How likely is it that you would recommend the vaccine to the patient?)	
Self-vaccination behaviour	Have you been vaccinated against Covid-19?	

Note. Text within brackets was adapted to the context in each country. Each contry version (including an English version) can be found at: https://osf.io/vha92/?view_only=dd330034e73d4aff901c4b4f5d60eecb. [a]Response scale: 1 = Strongly disagree; 2 = Somewhat disagree; 3 = Undecided; 4 = Somewhat agree; 5 = Strongly agree. [b]Not administered in Germany. [c]Text presented before the questions in the Perceived benefit-risk balance construct: “The following statements refer to the benefit/risk balance within the targeted population for each vaccine (e.g., the measles vaccine for infants).”

Table S3

Reliability Coefficients of Constructs

Construct	Reliability coefficient
Perceived vaccine risks	.92
Complacency	.83
Perceived benefit-risk balance	.82
Perceived collective responsibility	.80
Trust in authorities	.89
Commitment to vaccination	.74
Self-efficacy	.87
Openness to patients	.57
Perceived constraints	.63
Reluctant trust	.84
Professional norms	.93
Attitudes to mandates: Three items	.76
Attitudes to mandates: Two items	.78
Recommendation behavior	.67

Note. All coefficients are Cronbach's alpha, except for the constructs Perceived collective responsibility and Professional norms, which include only two items, and, therefore, the Spearman-Brown reliability coefficient was calculated.

Table S4

Distribution of the HCPs' Responses to the Questions on Attitudes to Mandates and Vaccination Attitudes

Item	Response	Finland		France		Germany		Portugal		Total	
		n	%	*n*	%	*n*	%	*n*	%	*n*	%
mand_hcp	1	18	4.64	46	3.77	61	10.05	114	19.66	239	8.55
	2	22	5.67	36	2.95	40	6.59	82	14.14	180	6.44
	3	17	4.38	62	5.08	43	7.08	68	11.72	190	6.80
	4	128	32.99	269	22.03	153	25.21	156	26.90	706	25.25
	5	203	52.32	808	66.18	310	51.07	160	27.59	1481	52.97
mand_public	1	66	17.01	105	8.60	83	13.67	146	25.17	400	14.31
	2	102	26.29	189	15.48	79	13.01	121	20.86	491	17.56
	3	72	18.56	275	22.52	99	16.31	83	14.31	529	18.92
	4	109	28.09	360	29.48	171	28.17	150	25.86	790	28.25
	5	39	10.05	292	23.91	175	28.83	80	13.79	586	20.96
mand_pass	1	15	3.87	95	7.78	-	-	44	7.59	154	7.03
	2	29	7.47	123	10.07	-	-	66	11.38	218	9.95
	3	34	8.76	237	19.41	-	-	81	13.97	352	16.07
	4	130	33.51	376	30.79	-	-	178	30.69	684	31.23
	5	180	46.39	390	31.94	-	-	211	36.38	781	35.71
safe_measles	1	1	0.26	17	1.39	0	0.00	1	0.17	19	0.68
	2	0	0.00	15	1.23	7	1.15	0	0.00	22	0.79
	3	2	0.52	21	1.72	9	1.48	2	0.34	34	1.22
	4	29	7.47	349	28.58	95	15.65	25	4.31	498	17.80
	5	356	91.75	819	67.08	496	81.87	552	95.17	2223	79.51
safe_flu	1	1	0.26	14	1.15	0	0.00	1	0.17	16	0.57
	2	0	0.00	12	0.98	9	1.48	1	0.17	22	0.79
	3	2	0.52	19	1.56	17	2.80	1	0.17	39	1.39
	4	28	7.22	309	25.31	125	20.59	43	7.41	505	18.06
	5	357	92.01	867	71.01	456	75.12	534	92.07	2214	79.18
safe_hepb	1	1	0.26	13	1.1	1	0.16	2	0.34	17	0.61
	2	0	0.00	15	1.2	2	0.33	1	0.17	18	0.64
	3	1	0.26	20	1.6	9	1.48	1	0.17	31	1.11
	4	20	5.15	336	27.5	93	15.32	17	2.93	466	16.67
	5	366	94.33	837	68.6	502	82.70	559	96.38	2264	80.97
safe_hpv	1	2	0.52	16	1.3	1	0.16	1	0.17	20	0.72
	2	1	0.26	17	1.4	6	0.99	0	0.00	24	0.86

	3	3	0.77	38	3.1	22	3.62	2	0.34	65	2.32
	4	42	10.82	372	30.5	131	21.58	26	4.48	571	20.42
	5	340	87.63	778	63.7	447	73.64	551	95.00	2116	75.68
comp_useful	1	293	75.52	816	66.8	349	57.50	508	87.59	1966	70.31
	2	79	20.36	280	22.9	142	23.39	39	6.72	540	19.31
	3	7	1.80	62	5.1	39	6.43	11	1.90	119	4.26
	4	7	1.80	53	4.3	56	9.23	19	3.28	135	4.83
	5	2	0.52	10	0.8	21	3.46	3	0.52	36	1.29
comp_many	1	336	86.60	970	79.4	396	65.24	528	91.03	2230	79.76
	2	46	11.86	169	13.8	126	20.76	24	4.14	365	13.05
	3	2	0.52	45	3.7	32	5.27	10	1.72	89	3.18
	4	2	0.52	28	2.3	36	5.93	13	2.24	79	2.83
	5	2	0.52	9	0.7	17	2.8	5	0.86	33	1.18
comp_young	1	333	85.82	1023	83.8	405	66.72	529	91.21	2290	81.90
	2	45	11.60	138	11.3	118	19.44	26	4.48	327	11.70
	3	8	2.06	35	2.9	40	6.59	7	1.21	90	3.22
	4	1	0.26	15	1.2	30	4.94	9	1.55	55	1.97
	5	1	0.26	10	0.8	14	2.31	9	1.55	34	1.22
balance_measles	1	1	0.26	3	0.25	1	0.16	0	0.00	5	0.18
	2	0	0.00	2	0.16	7	1.15	0	0.00	9	0.32
	3	2	0.52	6	0.49	16	2.64	2	0.34	26	0.93
	4	22	5.67	173	14.17	83	13.67	18	3.10	296	10.59
	5	363	93.56	1037	84.93	500	82.37	560	96.55	2460	87.98
balance_flu	1	1	0.26	2	0.16	4	0.66	2	0.34	9	0.32
	2	3	0.77	9	0.74	12	1.98	10	1.72	34	1.22
	3	3	0.77	64	5.24	34	5.60	11	1.90	112	4.01
	4	57	14.69	354	28.99	179	29.49	104	17.93	694	24.82
	5	324	83.51	792	64.86	378	62.27	453	78.10	1947	69.64
balance_hepb	1	1	0.26	2	0.16	3	0.49	2	0.34	8	0.29
	2	0	0.00	5	0.41	3	0.49	0	0.00	8	0.29
	3	2	0.52	23	1.88	15	2.47	2	0.34	42	1.50
	4	54	13.92	241	19.74	106	17.46	19	3.28	420	15.02
	5	331	85.31	950	77.81	480	79.08	557	96.03	2318	82.90
balance_hpv	1	1	0.26	2	0.16	3	0.49	0	0.00	6	0.21
	2	4	1.03	7	0.57	7	1.15	3	0.51	21	0.75
	3	3	0.77	76	6.22	29	4.78	4	0.69	112	4.01
	4	72	18.56	366	29.98	132	21.75	34	5.86	604	21.60
	5	308	79.38	770	63.06	436	71.83	539	92.93	2053	73.43
balance_cov	1	3	0.77	7	0.57	15	2.47	1	0.17	26	0.93

	2	2	0.52	12	0.98	10	1.65	7	1.21	31	1.11
	3	4	1.03	74	6.06	48	7.91	27	4.66	153	5.47
	4	82	21.13	333	27.27	146	24.05	126	21.72	687	24.57
	5	297	76.55	795	65.11	388	63.92	419	72.24	1899	67.92
coll_essential	1	2	0.52	7	0.57	7	1.15	1	0.17	17	0.61
	2	5	1.29	19	1.56	18	2.97	2	0.34	44	1.57
	3	8	2.06	57	4.67	25	4.12	3	0.52	93	3.33
	4	73	18.81	291	23.83	155	25.54	46	7.93	565	20.21
	5	300	77.32	847	69.37	402	66.23	528	91.03	2077	74.28
coll_explain	1	2	0.52	13	1.06	7	1.15	1	0.17	23	0.82
	2	5	1.29	40	3.28	16	2.64	7	1.21	68	2.43
	3	8	2.06	98	8.03	31	5.11	8	1.38	145	5.19
	4	95	24.48	406	33.25	180	29.65	59	10.17	740	26.47
	5	278	71.65	664	54.38	373	61.45	505	87.07	1820	65.09
trust_info	1	0	0.00	20	1.64	8	1.32	0	0.00	28	1.00
	2	1	0.26	33	2.70	19	3.13	3	0.52	56	2.00
	3	8	2.06	186	15.23	38	6.26	9	1.55	241	8.62
	4	54	13.92	619	50.70	202	33.28	90	15.52	965	34.51
	5	325	83.76	363	29.73	340	56.01	478	82.41	1506	53.86
trust_strategy	1	0	0.00	23	1.88	39	6.43	1	0.17	63	2.25
	2	2	0.52	48	3.93	79	13.01	9	1.55	138	4.94
	3	8	2.06	231	18.92	138	22.73	10	1.72	387	13.84
	4	86	22.16	606	49.63	224	36.90	149	25.69	1065	38.09
	5	292	75.26	313	25.63	127	20.92	411	70.86	1143	40.88
trust_safe	1	0	0.00	20	1.64	9	1.48	2	0.34	31	1.11
	2	1	0.26	35	2.87	17	2.80	5	0.86	58	2.07
	3	4	1.03	198	16.22	39	6.43	7	1.21	248	8.87
	4	55	14.18	594	48.65	185	30.48	89	15.34	923	33.01
	5	328	84.54	374	30.63	357	58.81	477	82.24	1536	54.94
commit_vax	1	3	0.77	1	0.82	3	0.49	0	0.00	7	0.25
	2	13	3.35	1	0.82	3	0.49	1	0.17	18	0.64
	3	48	12.37	20	1.64	15	2.47	3	0.52	86	3.08
	4	214	55.15	436	35.71	142	23.39	36	6.21	828	29.61
	5	110	28.35	763	62.49	444	73.15	540	93.10	1857	66.42
commit_knowledge	1	0	0.00	2	0.16	1	0.16	0	0.00	3	0.11
	2	1	0.26	6	0.49	1	0.16	1	0.17	9	0.32
	3	30	7.73	56	4.59	18	2.97	5	0.86	109	3.90
	4	189	48.71	526	43.08	160	26.36	46	7.93	921	32.94
	5	168	43.30	631	51.68	427	70.35	528	91.03	1754	62.73

commit_communic	1	0	0.00	17	1.39	5	0.82	0	0.00	22	0.79
	2	6	1.55	38	3.11	3	0.49	1	0.17	48	1.72
	3	28	7.22	206	16.87	17	2.80	8	1.38	259	9.26
	4	197	50.77	562	46.03	200	32.95	66	11.38	1025	36.66
	5	157	40.46	398	32.60	382	62.93	505	87.07	1442	51.57
effic_advice	1	1	0.26	1	0.08	0	0.00	1	0.17	3	0.11
	2	12	3.09	16	1.31	7	1.15	11	1.90	46	1.65
	3	13	3.35	67	5.49	24	3.95	16	2.76	120	4.29
	4	170	43.81	646	52.91	227	37.40	222	38.28	1265	45.24
	5	192	49.48	491	40.21	349	57.50	330	56.90	1362	48.71
effic_discuss	1	2	0.52	4	0.33	1	0.16	4	0.69	11	0.39
	2	21	5.41	59	4.83	11	1.81	18	3.10	109	3.90
	3	44	11.34	137	11.22	40	6.59	34	5.86	255	9.12
	4	187	48.20	595	48.73	242	39.87	281	48.45	1305	46.67
	5	134	34.54	426	34.89	313	51.57	243	41.90	1116	39.91
effic_traindisc	1	1	0.26	2	0.16	2	0.33	4	0.69	9	0.32
	2	19	4.90	23	1.88	9	1.48	16	2.76	67	2.40
	3	49	12.63	110	9.01	37	6.10	37	6.38	233	8.33
	4	206	53.09	640	52.42	241	39.70	331	57.07	1418	50.72
	5	113	29.12	446	36.53	318	52.39	192	33.10	1069	38.23
effic_trainbring	1	3	0.77	10	0.82	2	0.33	4	0.69	19	0.68
	2	26	6.70	50	4.10	19	3.13	24	4.14	119	4.26
	3	60	15.46	177	14.50	62	10.21	56	9.66	355	12.70
	4	217	55.93	615	50.37	242	39.87	311	53.62	1385	49.54
	5	82	21.13	369	30.22	282	46.46	185	31.90	918	32.83
open_legit	1	2	0.52	47	3.85	8	1.32	65	11.21	122	4.36
	2	31	7.99	210	17.20	83	13.67	193	33.28	517	18.49
	3	25	6.44	287	23.51	117	19.28	79	13.62	508	18.17
	4	201	51.80	512	41.93	249	41.02	196	33.79	1158	41.42
	5	130	33.25	165	13.51	150	24.71	47	8.10	491	17.56
open_influence	1	11	2.84	60	4.91	15	2.47	42	7.24	128	4.58
	2	126	32.47	275	22.52	69	11.37	117	20.17	587	20.99
	3	40	10.31	206	16.87	63	10.38	55	9.48	364	13.02
	4	156	40.21	480	39.31	263	43.33	210	36.21	1109	39.66
	5	55	14.18	200	16.38	197	32.45	156	26.90	608	21.75
open_delay	1	77	19.85	250	20.48	151	24.88	248	42.76	726	25.97
	2	198	51.03	505	41.36	220	36.24	222	38.28	1145	40.95
	3	44	11.34	196	16.05	95	15.65	27	4.66	362	12.95
	4	61	15.72	214	17.53	93	15.32	68	11.72	436	15.59

	5	8	2.06	56	4.59	48	7.91	15	2.59	127	4.54
const_cost	1	35	9.02	485	39.72	220	36.24	48	8.28	788	28.18
	2	89	22.94	253	20.72	183	30.15	58	10.00	583	20.85
	3	49	12.63	133	10.89	69	11.37	10	1.72	261	9.33
	4	172	44.33	272	22.28	106	17.46	302	52.07	852	30.47
	5	43	11.08	78	6.39	29	4.78	162	27.93	312	11.16
const_country	1	78	20.10	602	49.30	135	22.24	317	54.66	1132	40.49
	2	135	34.79	347	28.42	194	31.96	107	18.45	783	28.00
	3	95	24.48	132	10.81	85	14.00	48	8.28	360	12.88
	4	72	18.56	111	9.09	147	24.22	87	15.00	417	14.91
	5	8	2.06	29	2.38	46	7.58	21	3.62	104	3.72
const_practice	1	135	34.79	596	48.81	152	25.04	322	55.52	1205	43.10
	2	146	37.63	286	23.42	200	32.95	110	18.97	742	26.54
	3	65	16.75	145	11.88	82	13.51	40	6.90	332	11.87
	4	35	9.02	144	11.79	129	21.25	84	14.48	392	14.02
	5	7	1.80	50	4.10	44	7.25	24	4.14	125	4.47
reluct_inform	1	96	24.74	399	32.68	180	29.65	197	33.97	872	31.19
	2	120	30.93	416	34.07	226	37.23	133	22.93	895	32.01
	3	60	15.46	168	13.76	87	14.33	52	8.97	367	13.13
	4	90	23.20	181	14.82	92	15.16	148	25.52	511	18.28
	5	22	5.67	57	4.67	22	3.62	50	8.62	151	5.40
reluct_policy	1	115	29.64	401	32.84	160	26.36	219	37.76	895	32.01
	2	121	31.19	356	29.16	197	32.45	120	20.69	794	28.40
	3	75	19.33	188	15.40	108	17.79	73	12.59	444	15.88
	4	56	14.43	217	17.77	123	20.26	132	22.76	528	18.88
	5	21	5.41	59	4.83	19	3.13	36	6.21	135	4.83
reluct_safety	1	148	38.14	591	48.40	239	39.37	282	48.62	1260	45.06
	2	116	29.90	385	31.53	179	29.49	133	22.93	813	29.08
	3	68	17.53	127	10.40	75	12.36	59	10.17	329	11.77
	4	42	10.82	84	6.88	91	14.99	81	13.97	298	10.66
	5	14	3.61	34	2.78	23	3.79	25	4.31	96	3.43
norm_recom	1	1	0.26	1	0.08	0	0.00	1	0.17	3	0.11
	2	2	0.52	12	0.98	13	2.14	1	0.17	28	1.00
	3	4	1.03	84	6.88	41	6.75	16	2.76	145	5.19
	4	119	30.67	833	68.22	323	53.21	129	22.24	1404	50.21
	5	262	67.53	291	23.83	230	37.89	433	74.66	1216	43.49
norm_favour	1	1	0.26	1	0.08	0	0.00	2	0.34	4	0.14
	2	1	0.26	8	0.66	11	1.81	0	0.00	20	0.72
	3	3	0.77	89	7.29	36	5.93	12	2.07	140	5.01

4	114	29.38	815	66.75	332	54.70	110	18.97	1371	49.03
5	270	69.33	308	25.23	228	37.56	456	78.62	1261	45.10

Table S5

Mean Attitude to COVID-19 Vaccine Mandates

	Finland		France		Germany		Portugal		Total	
Variable	*M*	*SD*	*M*	*SD*	*M*	*SD*	*M*	*SD*	*M*	*SD*
HCP mandate	4.23	1.08	4.44	0.99	4.01	1.32	3.29	1.49	4.08	1.27
Public mandate	2.88	1.27	3.45	1.25	3.46	1.38	2.82	1.41	3.24	1.35
Health pass	4.11	1.09	3.69	1.23	-	-	3.77	1.26	3.79	1.23

Table S6

Results from Tukey's Tests

Variable	Comparison	Estimate	adjusted *p*
Attitudes to HCP mandates	Finland – France	0.21	.013
	Finland – Germany	-0.22	.024
	Finland – Portugal	-0.94	< .001
	France – Germany	-0.43	< .001
	France – Portugal	-1.15	< .001
	Germany – Portugal	-0.72	< .001
Attitudes to public mandates	Finland – France	0.57	< .001
	Finland – Germany	0.58	< .001
	Finland – Portugal	-0.06	.914
	France – Germany	0.01	.999
	France – Portugal	-0.62	< .001
	Germany – Portugal	-0.63	< .001
Attitudes to a health pass	Finland – France	-0.42	< .001
	Finland – Portugal	-0.34	< .001
	France – Portugal	0.08	.407

Table S7

Results from Regression Analyses with Attitudes to HCP Mandates, Attitudes to Public Mandates, and Attitudes to a Health Pass as Outcome Variable by Country

	HCP mandates			Public mandates			Health pass		
Fixed effects	*β*	*SE*	*p*	*β*	*SE*	*p*	*β*	*SE*	*p*
Finland									
Intercept	-0.00	0.05	1.000	-0.00	0.05	1.000	-0.00	0.05	1.000
Gender: Female vs male	-0.01	0.05	.861	-0.02	0.05	.706	-0.01	0.05	.875
Age: 40–49 vs < 40	-0.01	0.06	.846	0.02	0.06	.793	-0.00	0.06	.955
Age: 50– vs 40–49	0.12	0.06	.042	0.15	0.06	.011	0.14	0.06	.017
Vaccine safety	0.02	0.05	.750	-0.01	0.06	.920	-0.04	0.05	.461
Complacency	0.00	0.06	.941	0.08	0.06	.162	-0.05	0.06	.338
Benefit-risk balance	0.24	0.06	< .001	0.20	0.06	< .001	0.26	0.06	< .001
Collective responsibility	0.08	0.05	.096	0.06	0.05	.234	0.04	0.05	.454
Trust in authorities	0.11	0.06	.065	0.09	0.06	.128	0.19	0.06	.002
Commitment to vaccination	0.06	0.06	.286	0.16	0.06	.006	-0.03	0.06	.556
Self-efficacy	-0.00	0.06	.970	-0.00	0.06	.967	0.05	0.06	.385
Openness to patients	-0.16	0.05	.002	-0.08	0.05	.103	-0.06	0.05	.230
Constraints	0.04	0.05	.414	0.01	0.05	.765	0.01	0.05	.794
Reluctant trust	-0.12	0.05	.014	-0.05	0.05	.304	-0.02	0.05	.661
Professional norm	-0.08	0.05	.088	-0.04	0.05	.450	-0.02	0.05	.680
France									
Intercept	-0.00	0.02	.915	-0.00	0.03	.961	-0.00	0.03	.972
Gender: Female vs male	-0.02	0.03	.404	-0.02	0.03	.377	0.00	0.03	.928
Age: 40–49 vs < 40	0.07	0.03	.013	0.02	0.03	.586	0.06	0.03	.072
Age: 50– vs 40–49	0.01	0.03	.851	-0.01	0.03	.687	0.10	0.03	< .001
Vaccine safety	0.01	0.03	.741	-0.01	0.03	.603	-0.03	0.03	.240
Complacency	-0.10	0.03	< .001	0.00	0.03	.994	-0.04	0.03	.175
Benefit-risk balance	0.25	0.03	< .001	0.12	0.03	< .001	0.16	0.03	< .001
Collective responsibility	0.06	0.03	.017	0.13	0.03	< .001	0.04	0.03	.113
Trust in authorities	0.22	0.03	< .001	0.21	0.03	< .001	0.26	0.03	< .001
Commitment to vaccination	0.09	0.03	.002	0.10	0.03	.002	0.08	0.03	.007
Self-efficacy	-0.06	0.03	.039	0.01	0.03	.692	-0.04	0.03	.228
Openness to patients	-0.07	0.03	.007	-0.17	0.03	< .001	-0.09	0.03	.001
Constraints	0.06	0.03	.028	0.06	0.03	.039	-0.01	0.03	.826

Reluctant trust	-0.03	0.03	.229	0.01	0.03	.811	0.00	0.03	.906
Professional norm	-0.01	0.03	.709	0.01	0.03	.718	0.03	0.03	.250
			Germany						
Intercept	-0.00	0.03	.911	-0.00	0.04	.901	-	-	-
Gender: Female vs male	-0.06	0.03	.062	-0.05	0.04	.196	-	-	-
Age: 40–49 vs < 40	-0.04	0.04	.359	-0.07	0.04	.132	-	-	-
Age: 50– vs 40–49	0.10	0.04	.012	0.04	0.04	.383	-	-	-
Vaccine safety	0.03	0.05	.543	0.01	0.05	.821	-	-	-
Complacency	0.08	0.05	.062	0.03	0.05	.483	-	-	-
Benefit-risk balance	0.12	0.05	.033	0.05	0.06	.379	-	-	-
Collective responsibility	0.19	0.05	< .001	0.16	0.05	.001	-	-	-
Trust in authorities	0.34	0.05	< .001	0.29	0.05	< .001	-	-	-
Commitment to vaccination	-0.04	0.04	.392	0.00	0.05	.979	-	-	-
Self-efficacy	0.05	0.04	.223	0.06	0.05	.187	-	-	-
Openness to patients	-0.17	0.04	< .001	-0.12	0.04	.005	-	-	-
Constraints	0.02	0.04	.675	0.06	0.04	.147	-	-	-
Reluctant trust	0.01	0.04	.890	0.01	0.04	.858	-	-	-
Professional norm	-0.10	0.04	.005	-0.14	0.04	< .001	-	-	-
			Portugal						
Intercept	0.00	0.04	1.000	0.00	0.04	1.000	0.00	0.04	1.000
Gender: Female vs male	-0.03	0.04	.427	-0.02	0.04	.649	0.04	0.04	.335
Age: 40–49 vs < 40	-0.03	0.05	.558	-0.02	0.05	.751	-0.07	0.05	.197
Age: 50– vs 40–49	0.10	0.05	.044	0.05	0.05	.384	0.03	0.05	.546
Vaccine safety	-0.09	0.05	.057	-0.06	0.05	.210	-0.01	0.05	.746
Complacency	0.03	0.05	.493	0.06	0.05	.234	0.03	0.05	.506
Benefit-risk balance	0.16	0.05	.002	0.16	0.05	.002	0.15	0.05	.005
Collective responsibility	0.06	0.04	.179	0.05	0.04	.243	0.07	0.04	.113
Trust in authorities	0.02	0.05	.656	0.00	0.05	.930	0.08	0.05	.111
Commitment to vaccination	-0.02	0.05	.748	-0.02	0.05	.648	-0.01	0.05	.913
Self-efficacy	0.03	0.05	.464	0.05	0.05	.301	0.02	0.05	.627
Openness to patients	-0.16	0.04	< .001	-0.11	0.04	.012	-0.12	0.04	.006
Constraints	0.05	0.04	.248	0.08	0.04	.058	-0.01	0.04	.759
Reluctant trust	0.01	0.04	.869	0.03	0.04	.526	0.07	0.04	.121
Professional norm	-0.08	0.04	.050	-0.08	0.04	.081	0.05	0.04	.283

Figure S1. Response distribution of the recommendation behavior variable in each country.

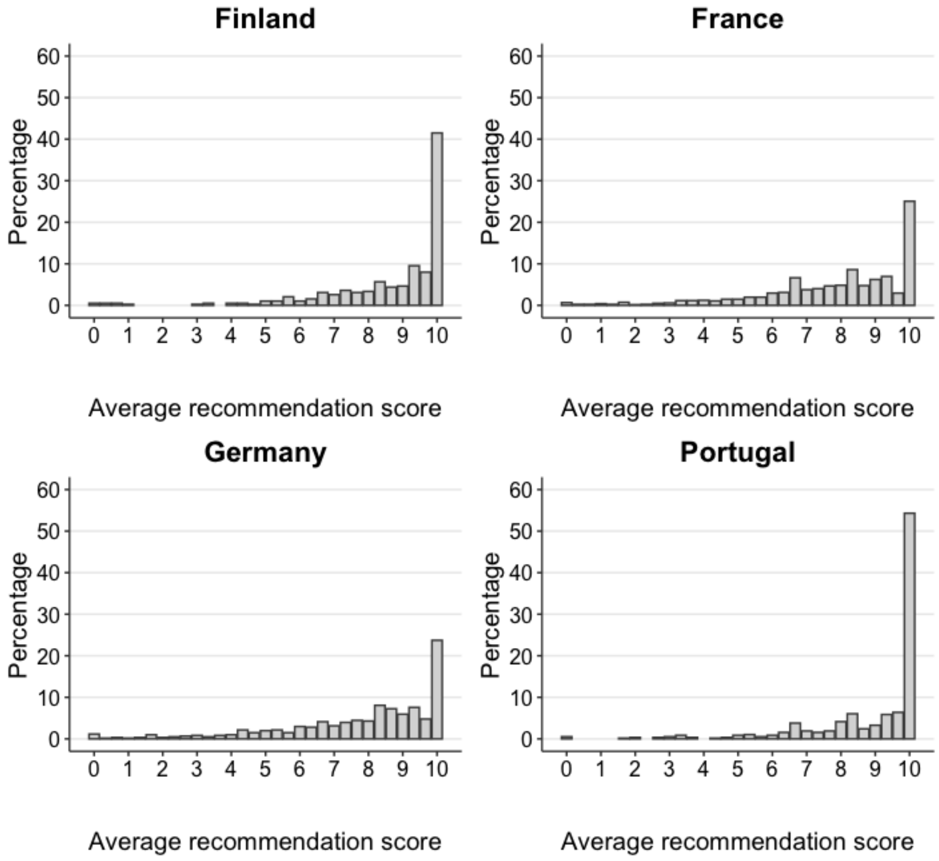

Supplement: Supplemental Material [file KHVI_A_2256442_SM9232.pdf]
